# Supplementary material for: Draft Genome Sequence of Priestia sp. Strain TSO9, a Plant Growth-Promoting Bacterium Associated with Wheat (Triticum turgidum subsp. durum) in the Yaqui Valley, Mexico
Source: Plants (Basel). 2022 Aug 28;11(17):2231. doi: 10.3390/plants11172231 (PMC9460074; doi:10.3390/plants11172231)
Supplement: Supplementary file 1 [file plants-11-02231-s001.zip › plants-1836313-supplementary.pdf]

Draft Genome Sequence of *Priestia* sp. Strain TSO9, a Plant Growth-Promoting Bacterium Associated with Wheat *(Triticum turgidum* subsp. *durum)* in the Yaqui Valley, Mexico

Maria Edith Ortega-Urquieta [1,†], Valeria Valenzuela-Ruíz [1,†], Debasis Mitra [2], Sajjad Hyder [3], Nabil I. Elsheery [4], Pradeep Kumar Das Mohapatra [2], Fannie Isela Parra-Cota [5,*] and Sergio de los Santos-Villalobos [1,*]

[1] Instituto Tecnológico de Sonora, 5 de febrero 818 sur, 85000 Ciudad Obregon, Mexico
[2] Department of Microbiology, Raiganj University, Raiganj 733 134, Uttar Dinajpur, West Bengal India, India
[3] Department of Botany, Government College Women University Sialkot, 51310 Sialkot, Pakistan
[4] Agricultural Botany Department, Faculty of Agriculture, Tanta University, Tanta 31527, Egypt
[5] Campo Experimental Norman E. Borlaug, Instituto Nacional de Investigaciones Forestales, Agrícolas y Pecuarias, Ciudad Obregon P.C. 85000, Sonora, Mexico
* Correspondence: parra.fannie@inifap.gob.mx (F.I.P.-C.); sergio.delossantos@itson.edu.mx (S.d.l.S.-V.)
† These authors contributed equally to this work.

Table S1. Features of strain TSO9 genome by RAST (bacteriocins and ribosomally synthesized antibacterial peptides, resistance to antibiotics and toxic compounds, invasion and intracellular resistance, auxin biosynthesis, phosphorous metabolism, siderophores, osmotic stress response, and oxidative stress response).

Protein	Function
	**Bacteriocins and ribosomally synthesized antibacterial peptides**
BceB	Bacitracin export permease protein BceB
BceA	Bacitracin export ATP-binding protein BceA
BceS	Two-component sensor histidine kinase BceS
BceR	Two-component response regulator BceR
LiaR	Response regulator LiaR
LiaS	Sensor histidine kinase LiaS
LiaF	Transporter LiaF
LiaG	Conserved protein LiaG in B. subtilis in Lia cluster
LiaH	Protein LiaH, similar to phage shock protein A
LiaI	Protein LiaI
YvcP	Two-component response regulator YvcP
YvcQ	Two-component sensor kinase YvcQ
YvcR	ABC transporter ATP-binding protein YvcR
YvcS	ABC transporter permease protein YvcS

	**Resistance to antibiotics and toxic compounds**
CRTR	Cu(I)-responsive transcriptional regulator
CIA	Copper-translocating P-type ATPase (EC 3.6.3.4)
CopZ	Copper chaperone
HP	hypothetical protein PFL_0711
ClfA	Multidrug resistance transporter, Bcr/CflA family
MO	Multicopper oxidase
BCO	Blue copper oxidase CueO precursor
CT	Copper tolerance protein
Cc	Cytochrome c family protein
Cc1	FIG002261: Cytochrome c family protein
Cc2	FIG135464: Cytochrome c4
p553	Putative diheme cytochrome c-553
CBPP	Copper-binding periplasmic protein
PCLP	putative cytochrome C-like protein
ErfK	ErfK/YbiS/YcfS/YnhG family protein
Ccml	Cytochrome c heme lyase subunit CcmL
cycH	Cytochrome c-type biogenesis protein CycH
CcmG	Cytochrome c-type biogenesis protein CcmG/DsbE, thiol: disulfide oxidoreductase
Ccsp	Cytochrome c-type synthesis protein
CRD	Copper resistance protein D
CopC	Copper resistance protein CopC
CopD	Copper resistance protein CopD
CopG	CopG protein
CcmH	Cytochrome c heme lyase subunit CcmH
CcmF	Cytochrome c heme lyase subunit CcmF
Cbp	Cytochrome c-type biogenesis protein, putative
CRB	Copper resistance protein B
CSA	Copper/silver efflux P-type ATPase
CcmE	Cytochrome c-type biogenesis protein CcmE, heme chaperone
TR	Negative transcriptional regulator-copper transport operon
copS	Sensor protein copS (EC 2.7.3.-)
copR	Transcriptional activator protein CopR
copCp	Copper resistance protein C precursor
CusS?	Heavy metal sensor histidine kinase
CusR?	DNA-binding heavy metal response regulator
CusS	Copper sensory histidine kinase CusS
CusR	Copper-sensing two-component system response regulator CusR
2?	Heavy metal-(Cd/Co/Hg/Pb/Zn)-translocating P-type ATPase: Heavy metal translocating P-type ATPase

CZCR	Cobalt-zinc-cadmium resistance protein
czcD	Cobalt-zinc-cadmium resistance protein CzcD
zitB	Zinc transporter ZitB
DMT	Permease of the drug/metabolite transporter (DMT) superfamily
HGH	Hydroxyacylglutathione hydrolase (EC 3.1.2.6)
Dps	Non-specific DNA-binding protein Dps / Iron-binding ferritin-like antioxidant protein / Ferroxidase (EC 1.16.3.1)
HNA	Hypothetical N-acetyltransferase
TA	Trans-aconitate 2-methyltransferase (EC 2.1.1.144)
DMT2	Putative DMT superfamily metabolite efflux protein precursor
RtK	adherence and invasion membrane-associated protein (Ail, less powerful adhesin than Inv, provides resistance to killing by serum complement)
YPO	Hypothetical protein YPO2504
pnuCl	Ribosyl nicotinamide transporter, PnuC-like
QS	Quinolinate synthetase (EC 2.5.1.72)
CzcA	Cobalt-zinc-cadmium resistance protein CzcA
CzcC	Heavy metal RND efflux outer membrane protein, CzcC family
SEP	Putative silver efflux pump
cusB	Putative copper efflux system protein CusB
FP	Probable Co/Zn/Cd efflux system membrane fusion protein
CzcB	Cobalt/zinc/cadmium efflux RND transporter, membrane fusion protein, CzcB family
nccB	Nickel-cobalt-cadmium resistance protein NccB
cusA	Cation efflux system protein CusA
CzrB	Zn(II) and Co(II) transmembrane diffusion facilitator
HME	Hypothetical protein involved in heavy metal export
CzrR	DNA-binding heavy metal response regulator
HMHK	Heavy metal sensor histidine kinase
CusR	Copper-sensing two-component system response regulator CusR
CusS	Copper sensory histidine kinase CusS
cusF	Cation efflux system protein CusF precursor
cusC	Cation efflux system protein CusC precursor
TRCd	Cd(II)/Pb(II)-responsive transcriptional regulator
TRMer	Transcriptional regulator, MerR family
HmrR	Heavy metal resistance transcriptional regulator HmrR
nccA	Nickel-cobalt-cadmium resistance protein NccA
PAs	P pilus assembly/Cpx signaling pathway, periplasmic inhibitor/zinc-resistance associated protein
PCT	Probable cadmium-transporting ATPase (EC 3.6.3.3)
CRA	Cadmium-transporting ATPase (EC 3.6.3.3)
DcyD	D-cysteine desulfhydrase (EC 4.4.1.15)
CBP	L-Cystine ABC transporter, periplasmic cystine-binding protein

CPP	Cystine ABC transporter, permease protein
CAP	Cystine ABC transporter, ATP-binding protein
ZraS	Sensor protein of zinc sigma-54-dependent two-component system
ZraR	Response regulator of zinc sigma-54-dependent two-component system
ZraP	Zinc resistance-associated protein
ScsA	Suppression of copper sensitivity: putative copper-binding protein ScsA
ScsB	Membrane protein, suppressor for copper-sensitivity ScsB
ScsC	Secreted protein, suppressor for copper-sensitivity ScsC
ScsD	Membrane protein, suppressor for copper-sensitivity ScsD
CutA	Periplasmic divalent cation tolerance protein CutA
CutC	Cytoplasmic copper homeostasis protein CutC
CutF	Copper homeostasis protein CutF precursor
CutE	Copper homeostasis protein CutE
CorC	Magnesium and cobalt efflux protein CorC
FosA	Fosfomycin resistance protein FosA
FosB	Fosfomycin resistance protein FosB
FosX	Fosfomycin resistance protein FosX
parC	Topoisomerase IV subunit A (EC 5.99.1.-)
parE	Topoisomerase IV subunit B (EC 5.99.1.-)
gyrA	DNA gyrase subunit A (EC 5.99.1.3)
gyrB	DNA gyrase subunit B (EC 5.99.1.3)
Lde	Efflux pump Lde
BL	Beta-lactamase (EC 3.5.2.6)
BLc	Beta-lactamase class C and other penicillin-binding proteins
BLI	Metal-dependent hydrolases of the beta-lactamase superfamily I
BLR	Negative regulator of beta-lactamase expression
bl	Beta-lactamase
BLII	Metal-dependent hydrolases of the beta-lactamase superfamily II
BLIII	Metal-dependent hydrolases of the beta-lactamase superfamily III
BLA	Beta-lactamase class A
CTX	Beta-lactamase CTX-M-16
blaR	Regulatory protein BlaR1
blaI	Beta-lactamase repressor BlaI
BLD	Beta-lactamase class D
OXA	Beta-lactamase OXA-18 precursor (EC 3.5.2.6)
AmpS	Beta-lactamase AmpS
PSE2	Beta-lactamase PSE-2 precursor (EC 3.5.2.6)
ybxl	Probable beta-lactamase ybxI precursor (EC 3.5.2.6)
CS	Beta-lactamase (Cephalosporinase) (EC 3.5.2.6)
cadA	Cadmium-transporting ATPase (EC 3.6.3.3)
cadC	Cadmium efflux system accessory protein

cadD	Cadmium resistance protein
CmeA	RND efflux system, membrane fusion protein CmeA
CmeB	RND efflux system, inner membrane transporter CmeB
CmeC	RND efflux system, outer membrane lipoprotein CmeC
AcrR	Transcription repressor of multidrug efflux pump acrAB operon, TetR (AcrR) family
TetR	Transcription regulator of multidrug efflux pump operon, TetR (AcrR) family
Reg	Probable transcription regulator protein of MDR efflux pump cluster
MATE_all	Multi antimicrobial extrusion protein (Na(+)/drug antiporter), MATE family of MDR efflux pumps
YdhE/NorM	Multidrug and toxin extrusion (MATE) family efflux pump YdhE/NorM
YdhE/NorM?	Multidrug and toxin extrusion (MATE) family efflux pump YdhE/NorM, homolog
MFS	Multidrug-efflux transporter, major facilitator superfamily (MFS) (TC 2.A.1)
VNT	Vesicular neurotransmitter transporter
MacA	Macrolide-specific efflux protein MacA
MacB	Macrolide export ATP-binding/permease protein MacB (EC 3.6.3.-)
OML	RND efflux system, outer membrane lipoprotein, NodT family
TolC_14	Type I secretion outer membrane protein, TolC precursor
MtrF	Multidrug efflux pump component MtrF
RND	Membrane fusion protein of RND family multidrug efflux pump
AcrB	Acriflavin resistance protein
OM	Probable outer membrane component of multidrug efflux pump
MexD	Multidrug efflux RND transporter MexD
MexC	Multidrug efflux RND membrane fusion protein MexC
NfxB	Transcriptional regulator NfxB
PmrA	Multidrug resistance efflux pump PmrA
	**Invasion and intracellular resistance**
Rv0682	SSU ribosomal protein S12p (S23e)
Rv0683	SSU ribosomal protein S7p (S5e)
Rv0684	Translation elongation factor G
Rv0685	Translation elongation factor Tu
Rv0686	FIG025093: Probable membrane protein
Rv0666	FIG108202: hypothetical protein
Rv0667	DNA-directed RNA polymerase beta subunit (EC 2.7.7.6)
Rv0668	DNA-directed RNA polymerase beta' subunit (EC 2.7.7.6)
Rv1594	Quinolinate synthetase (EC 2.5.1.72)
Rv1595	L-aspartate oxidase (EC 1.4.3.16)
Rv1596	Quinolinate phosphoribosyltransferase [decarboxylating] (EC 2.4.2.19)
Rv1641	Translation initiation factor 3

Rv1642	LSU ribosomal protein L35p
Rv1643	LSU ribosomal protein L20p

Auxin biosynthesis

APRT	Anthranilate phosphoribosyltransferase (EC 2.4.2.18)
PRAI	Phosphoribosylanthranilate isomerase (EC 5.3.1.24)
IGS	Indole glycerophosphate synthase
TSa	Tryptophan synthase alpha chain (EC 4.2.1.20)
TSb	Tryptophan synthase beta chain (EC 4.2.1.20)
TM	tryptophan monooxygenase
IAH	Indole-acetamide hydrolase
N3	Nitrilase 1 (EC 3.5.5.1)
TT	Tryptophan transaminase
IPAC	Indole-3-pyruvate decarboxylase (EC 4.1.1.74)
IAD	Indole-acetaldehyde dehydrogenase
AAD	Aromatic-L-amino-acid decarboxylase (EC 4.1.1.28)
AO	Monoamine oxidase (1.4.3.4)
IAR	Indole-acetaldehyde reductase
TO	Tryptophol oxidase
BoundA	Indole-3-acetate beta-glucosyltransferase
ATFS	Flavonol 3-sulfotransferase (EC 2.8.2.25)
N2	Nitrilase 2 (EC 3.5.5.1)
AUX1	Putative AUX1-like permease

Phosphorus metabolism

PepM	Phosphoenolpyruvate phosphomutase (EC 5.4.2.9)
deCO2	Phosphonopyruvate decarboxylase (EC 4.1.1.82)
2-APAT	2-aminoethylphosphonate:pyruvate aminotransferase (EC 2.6.1.37)
SPAT	Serine-pyruvate aminotransferase/archaeal aspartate aminotransferase
PhoU?	Distant similarity with phosphate transport system regulator
PhoU	Phosphate transport system regulatory protein PhoU
pstS	Phosphate ABC transporter, periplasmic phosphate-binding protein PstS (TC 3.A.1.7.1)
PstS?	Putative periplasmic phosphate-binding protein PstS (Mycoplasma type)
PstS_hal	Putative periplasmic phosphate-binding protein PstS (Halobacteriales type)
PstS_c	Putative periplasmic phosphate-binding protein PstS (Catenulisporaceae type)
PstA	Phosphate transport system permease protein PstA (TC 3.A.1.7.1)

PstB	Phosphate transport ATP-binding protein PstB (TC 3.A.1.7.1)
PstC	Phosphate transport system permease protein PstC (TC 3.A.1.7.1)
PhoP	Alkaline phosphatase synthesis transcriptional regulatory protein PhoP
PhoR	Phosphate regulon sensor protein PhoR (SphS) (EC 2.7.13.3)
PhoB	Phosphate regulon transcriptional regulatory protein PhoB (SphR)
PPiK	Polyphosphate kinase (EC 2.7.4.1)
PPiase	Exopolyphosphatase (EC 3.6.1.11)
OprO	Pyrophosphate-specific outer membrane porin OprO
OprP	Phosphate-specific outer membrane porin OprP
DrrA_PhoP	response regulator DrrA
PstSp??	Putative phosphate ABC transporter, phosphate-binding component
IPP	Inorganic pyrophosphatase (EC 3.6.1.1)
THb	NAD(P) transhydrogenase subunit beta (EC 1.6.1.2)
THa	NAD(P) transhydrogenase alpha subunit (EC 1.6.1.2)
PhoHv	Phosphate starvation-inducible protein PhoH, predicted ATPase
SAP	secreted alkaline phosphatase
phoP	Alkaline phosphatase synthesis transcriptional regulatory protein PhoP
phoQ	response regulator in two-component regulatory system with PhoQ
TH2	Soluble pyridine nucleotide transhydrogenase (EC 1.6.1.1)
ASGA	1-acyl-sn-glycerol-3-phosphate acyltransferase (EC 2.3.1.51)
YggT	Integral membrane protein YggT, involved in response to extracytoplasmic stress (osmotic shock)
ALPT	Apolipoprotein N-acyltransferase (EC 2.3.1.-)
corC	Magnesium and cobalt efflux protein CorC
MH	Metal-dependent hydrolase YbeY, involved in rRNA and/or ribosome maturation and assembly
MDIP	Manganese-dependent inorganic pyrophosphatase (EC 3.6.1.1)
AP	Alkaline phosphatase (EC 3.1.3.1)
PhoQ	PhoQ
LAT	Low-affinity inorganic phosphate transporter
PLAT	Probable low-affinity inorganic phosphate transporter

PhoUh	Phosphate transport regulator (distant homolog of PhoU)
PPK	Polyphosphate kinase (EC 2.7.4.1)
EPP	Exopolyphosphatase (EC 3.6.1.11)
GDP	Guanosine-5'-triphosphate,3'-diphosphate pyrophosphatase (EC 3.6.1.40)
PhoU	Phosphate transport system regulatory protein PhoU
NaPi	Sodium-dependent phosphate transporter
APl	Alkaline phosphatase like protein
AP?	putative alkaline phosphatase-like protein
PhoH?	Predicted ATPase related to phosphate starvation-inducible protein PhoH
EPP?GPP?	Phosphatase, Ppx/GppA family
PEPP	Pyrophosphate-energized proton pump (EC 3.6.1.1)
PhoR	Phosphate regulon sensor protein PhoR (SphS) (EC 2.7.13.3)
PhoB	Phosphate regulon transcriptional regulatory protein PhoB (SphR)
PhoU?	Distant similarity with phosphate transport system regulator
ptsS	Phosphate ABC transporter, periplasmic phosphate-binding protein PstS (TC 3.A.1.7.1)
ptsS?	Putative periplasmic phosphate-binding protein PstS (Mycoplasma type)
ptsA	Phosphate transport system permease protein PstA (TC 3.A.1.7.1)
ptsC	Phosphate transport system permease protein PstC (TC 3.A.1.7.1)
ptsB	Phosphate transport ATP-binding protein PstB (TC 3.A.1.7.1)
OprO	Pyrophosphate-specific outer membrane porin OprO
OprP	Phosphate-specific outer membrane porin OprP
PpaX	Inorganic pyrophospatase PpaX (EC 3.1.3.18)
ET1	Phosphate permease PHO89 (Na( )/Pi cotransporter PHO89)
PpaX2	Pyrophosphatase PpaX (EC 3.6.1.1)
Ppx	Exopolyphosphatase (EC 3.6.1.11)
Ppk	Polyphosphate kinase (EC 2.7.4.1)
Ppk2	Polyphosphate kinase 2 (EC 2.7.4.1)
Gdp	Guanosine-5'-triphosphate,3'-diphosphate pyrophosphatase (EC 3.6.1.40)
PPGK	Polyphosphate glucokinase (EC 2.7.1.63)

PPN1	Endopolyphosphatase (EC 3.6.1.10)

Siderophores

Sdab	Siderophore biosynthesis diaminobutyrate--2-oxoglutarate aminotransferase (EC 2.6.1.76)
Sdad	Siderophore biosynthesis L-2,4-diaminobutyrate decarboxylase
SSlAc	Siderophore synthetase large component, acetyltransferase
SSsAc	Siderophore synthetase small component, acetyltransferase
SSLc	Siderophore synthetase component, ligase
SbsM	Siderophore biosynthesis protein, monooxygenase
LCFAL	Long-chain-fatty-acid--CoA ligase of siderophore biosynthesis
STra	Siderophore transport protein
STonBrec	TonB-dependent siderophore receptor
FeSRTon	Ferric siderophore receptor, TonB dependent
Sperm	Siderophore related permease
FhuA	Ferric hydroxamate outer membrane receptor FhuA
FhuB	Ferric hydroxamate ABC transporter (TC 3.A.1.14.3), permease component FhuB
FhuC	Ferric hydroxamate ABC transporter (TC 3.A.1.14.3), ATP-binding protein FhuC
FhuD	Ferric hydroxamate ABC transporter (TC 3.A.1.14.3), periplasmic substrate binding protein FhuD
FeSB	Iron-siderophore transport system, substrate-binding component
FeTM	Iron-siderophore transport system, transmembran component
FeP	Iron-siderophore transport system, permease component
FeATP	Iron-siderophore transport system, ATP-binding component
PchR	Transcriptional regulator PchR
PchA	Isochorismate synthase (EC 5.4.4.2) of siderophore biosynthesis
PchB	Isochorismate pyruvate-lyase (EC 4.-.-.-) of siderophore biosynthesis
PchC	Thioesterase in siderophore biosynthesis gene cluster
PchD	2,3-dihydroxybenzoate-AMP ligase (EC 2.7.7.58) of siderophore biosynthesis

DHBAMPL1	2,3-dihydroxybenzoate-AMP ligase (EC 2.7.7.58)
PchEPchF	Siderophore biosynthesis non-ribosomal peptide synthetase modules
PchG	Thiazolinyl imide reductase in siderophore biosynthesis gene cluster
PchHPchI	Putative ABC iron siderophore transporter, fused permease and ATPase domains
CycPep	ABC-type siderophore export system, fused ATPase and permease components
FptA	Outer membrane receptor for ferric siderophore
FtpB	Hypothetical protein FtpB in siderophore gene cluster
FtpC	Putative iron-regulated membrane protein in siderophore cluster
FptX	Inner-membrane permease for ferric siderophore
ICM	Isochorismatase (EC 3.3.2.1) of siderophore biosynthesis
BDH	2,3-dihydro-2,3-dihydroxybenzoate dehydrogenase (EC 1.3.1.28) of siderophore biosynthesis
FeR	Ferric reductase (1.6.99.14)
PchK	Putative reductoisomerase in siderophore biosynthesis gene cluster
ABCp	ABC-type Fe3+-siderophore transport system, permease component
ABCatp	ABC-type Fe3+-siderophore transport system, ATPase component
ABCsb	ABC-type Fe3+-siderophore transport system, periplasmic iron-binding component
ABCp2	ABC-type Fe3+-siderophore transport system, permease 2 component
Tonhem	TonB-dependent hemin receptor
hmuU	Hemin ABC transporter, permease protein
hmuV	ABC-type hemin transport system, ATPase component
hmuT	Periplasmic hemin-binding protein
hmuS	Hemin transport protein HmuS
hmuR	TonB-dependent hemin , ferrichrome receptor
estlip	Esterase/lipase in siderophore cluster
Hyp1	Antibiotic biosynthesis monooxygenase domain-containing protein
AsbA	Anthrachelin biosynthesis protein AsbA
AsbB	Anthrachelin biosynthesis protein AsbB

SiderX3	Long-chain-fatty-acid--CoA ligase associated with anthrachelin biosynthesis
SiderX4	Hypothetical protein GBAA1985 associated with anthrachelin biosynthesis, unique
SiderX5	Hypothetical protein GBAA1986 associated with anthrachelin biosynthesis, isomerase-like TIM barrel domain
SiderX6	Acyl carrier protein associated with anthrachelin biosynthesis
X-ABC1	Uncharacterized iron compound ABC uptake transporter, substrate-binding protein
X-ABC2	Uncharacterized iron compound ABC uptake transporter, permease protein
X-ABC3	Uncharacterized iron compound ABC uptake transporter, ATP-binding protein
Fe-ABC1	Iron compound ABC uptake transporter substrate-binding protein
Fe-ABC2	Iron compound ABC uptake transporter permease protein
	**Osmotic stress response**
Aqua	Aquaporin Z
Glycerol	Glycerol uptake facilitator protein
Prop(OH)2	Propanediol diffusion facilitator
OmpA	Outer membrane protein A precursor
OsmY	Osmotically inducible protein OsmY
BetA	Choline dehydrogenase (EC 1.1.99.1)
BetB	Betaine aldehyde dehydrogenase (EC 1.2.1.8)
BetT	High-affinity choline uptake protein BetT
BetC	Choline-sulfatase (EC 3.1.6.6)
BetI	HTH-type transcriptional regulator BetI
OpuD	Glycine betaine transporter OpuD
OpuAA	Glycine betaine ABC transport system, ATP-binding protein OpuAA (EC 3.6.3.32)
OpuAB	Glycine betaine ABC transport system, permease protein OpuAB
OpuAC	Glycine betaine ABC transport system, glycine betaine-binding protein OpuAC
OpuABC	Glycine betaine ABC transport system, permease/glycine betaine-binding

	protein OpuABC
OpuC	Glycine betaine/L-proline ABC transporter, glycine betaine/L-proline-binding/permease protein
ProX	L-proline glycine betaine binding ABC transporter protein ProX (TC 3.A.1.12.1)
ProV	L-proline glycine betaine ABC transport system permease protein ProV (TC 3.A.1.12.1)
ProW	L-proline glycine betaine ABC transport system permease protein ProW (TC 3.A.1.12.1)
ProZ	Glycine betaine ABC transport system permease protein
CodA	Choline oxidase (E.C. 1.1.3.17)
OpuBA	Choline ABC transport system, ATP-binding protein OpuBA
OpuBB	Choline ABC transport system, permease protein OpuBB
OpuBC	Choline ABC transport system, choline-binding protein OpuBC
OpuBD	Choline ABC transport system, permease protein OpuBD
GbsB	Alcohol dehydrogenase GbsB (type III), essential for the utilization of choline (EC 1.1.1.1)
OpuCC	Osmotically activated L-carnitine/choline ABC transporter, substrate-binding protein OpuCC
OpuCD	Osmotically activated L-carnitine/choline ABC transporter, permease protein OpuCD
OpuCB	Osmotically activated L-carnitine/choline ABC transporter, permease protein OpuCB
OpuCA	Osmotically activated L-carnitine/choline ABC transporter, ATP-binding protein OpuCA
SoxA	Sarcosine oxidase alpha subunit (EC 1.5.3.1)
SoxB	Sarcosine oxidase beta subunit (EC 1.5.3.1)
SoxD	Sarcosine oxidase delta subunit (EC 1.5.3.1)
SoxG	Sarcosine oxidase gamma subunit (EC 1.5.3.1)
ChD	Choline binding protein D
ChA	Choline binding protein A
GbcA	GbcA Glycine betaine demethylase subunit A
GbcB	GbcB Glycine betaine demethylase subunit B

Oxidative stress response

sodA	Manganese superoxide dismutase (EC 1.15.1.1)
sodB	Superoxide dismutase [Fe] (EC 1.15.1.1)
sodC	Superoxide dismutase [Cu-Zn] precursor (EC 1.15.1.1)
HPII	Catalase (EC 1.11.1.6)
HPI	Peroxidase (EC 1.11.1.7)
CCP	Cytochrome c551 peroxidase (EC 1.11.1.5)
NSTR	Nitrite-sensitive transcriptional repressor NsrR
BphO	bacteriophytochrome heme oxygenase BphO
PCh	Phytochrome, two-component sensor histidine kinase (EC 2.7.3.-)
IBP	Iron-binding ferritin-like antioxidant protein
Dps	Non-specific DNA-binding protein Dps
Fr	Ferroxidase (EC 1.16.3.1)
PRP	DNA binding protein starved cells-like peroxide resistance protein
Rex	Redox-sensitive transcriptional regulator (AT-rich DNA-binding protein)
SoxR	Redox-sensitive transcriptional activator SoxR
SoxS	Regulatory protein SoxS
OxyR	Hydrogen peroxide-inducible genes activator
Furp	Ferric uptake regulation protein
Fnr	Fumarate and nitrate reduction regulatory protein
Furl	transcriptional regulator, Fur family
FeZn	Fe2+/Zn2+ uptake regulation proteins
Crp	transcriptional regulator, Crp/Fnr family
NnrS	NnrS protein involved in response to NO
MTN	Metallothionein
PIPA	Paraquat-inducible protein A
PIPB	Paraquat-inducible protein B
AhpC	Alkyl hydroperoxide reductase subunit C-like protein
PerR	Peroxide stress regulator
HPII	Catalase HPII (EC 1.11.1.6)

HPI_24	Peroxidase/catalase HPI (EC 1.11.1.6)
C22	Alkyl hydroperoxide reductase C22 protein (EC 1.8.1.-)
TR	Transcriptional regulator, Crp/Fnr family
FUR2	Transcriptional regulator, FUR family
Irr	Iron-responsive regulator Irr
ZUR	Zinc uptake regulation protein ZUR
FUR	Ferric uptake regulation protein FUR
PerR2	Peroxide stress regulator PerR, FUR family
Osmcl	Organic hydroperoxide resistance transcriptional regulator
OsmclR	Organic hydroperoxide resistance protein
HPIIb	Catalase (EC 1.11.1.6)
HPI_35	Peroxidase (EC 1.11.1.7)
SODA	Manganese superoxide dismutase (EC 1.15.1.1)
SODB	Superoxide dismutase [Fe] (EC 1.15.1.1)
SODC	Superoxide dismutase [Cu-Zn] precursor (EC 1.15.1.1)
RTH	Rubrerythrin
SOR	Superoxide reductase (EC 1.15.1.2)
SODFe/Mn	Superoxide dismutase [Mn/Fe] (EC 1.15.1.1)
SODMn	Superoxide dismutase [Mn] (EC 1.15.1.1)
SOD?	Superoxide dismutase (EC 1.15.1.1)
SODFe/Zn	superoxide dismutase [Fe-Zn] (EC 1.15.1.1)
HemO	Heme oxygenase HemO, associated with heme uptake
TP	Thioredoxin peroxidase 1 (EC 1.6.4.-)
PR	Probable peroxiredoxin (EC 1.11.1.15)
GT_a	Glutathione S-transferase, alpha (EC 2.5.1.18)
GT_d	Glutathione S-transferase, delta (EC 2.5.1.18)
GT_e	Glutathione S-transferase, epsilon (EC 2.5.1.18)
GT_f	Glutathione S-transferase, phi (EC 2.5.1.18)
GT_k	Glutathione S-transferase, kappa (EC 2.5.1.18)
GT_m	Glutathione S-transferase, mu (EC 2.5.1.18)

GT_om	Glutathione S-transferase, omega (EC 2.5.1.18)
GT_p	Glutathione S-transferase, pi (EC 2.5.1.18)
GT_t	Glutathione S-transferase, theta (EC 2.5.1.18)
GT_z	Glutathione S-transferase, zeta (EC 2.5.1.18)
GT_u	Glutathione S-transferase, unnamed subgroup (EC 2.5.1.18)
GT_u2	Glutathione S-transferase, unnamed subgroup 2 (EC 2.5.1.18)
GT_St	Glutathione S-transferase, Streptococcal type (EC 2.5.1.18)
GST	Glutathione S-transferase (EC 2.5.1.18)
YfcF	Probable glutathione S-transferase (EC 2.5.1.18), YfcF homolog
YfcG	Probable glutathione S-transferase (EC 2.5.1.18), YfcG homolog
Hyp1	Uncharacterized glutathione S-transferase-like protein
Hyp3	Distant similarity with leukotriene C4 synthase (microsomal glutathione S-transferase)
YncG	Uncharacterized GST-like protein yncG
YibF	Uncharacterized GST-like protein yibF
YghU	Uncharacterized GST-like protein yghU associated with glutathionylspermidine synthetase/amidase
Hyp4	Glutathione S-transferase family protein
MGS	Methylglyoxal synthase (EC 4.2.3.3)
GloA	Lactoylglutathione lyase (EC 4.4.1.5)
GloB	Hydroxyacylglutathione hydrolase (EC 3.1.2.6)
SAM1	FIG005121: SAM-dependent methyltransferase (EC 2.1.1.-)
SAM2	SAM-dependent methyltransferase 2, in cluster with Hydroxyacylglutathione hydrolase (EC 3.1.2.6)
GloB?	Similar to Hydroxyacylglutathione hydrolase, but in an organism lacking glutathione biosynthesis
PCS_b	Phytochelatin synthase (EC 2.3.2.15), bacterial type
PCS_e	Phytochelatin synthase (EC 2.3.2.15), eukaryotic type
GshA	Glutamate--cysteine ligase (EC 6.3.2.2)
GshAe	Glutamate--cysteine ligase eukaryotic (EC 6.3.2.2)
GshAd	Glutamate--cysteine ligase (EC 6.3.2.2), divergent, of Alpha- and Beta-proteobacteria type

GshB	Glutathione synthetase (EC 6.3.2.3)
GshBd	Predicted alternative glutathione synthetase (EC 6.3.2.3)
AB	Glutathione biosynthesis bifunctional protein gshF (EC 6.3.2.2)(EC 6.3.2.3)
YcbL	Hypothetical metal-binding enzyme, YcbL homolog
GR	Glutathione reductase (EC 1.8.1.7)
GPX	Glutathione peroxidase (EC 1.11.1.9)
GRX1	Glutaredoxin 1
GRX2	Glutaredoxin 2
GRX3	Glutaredoxin 3
GRX31	Glutaredoxin 3 (Grx1)
GRX32	Glutaredoxin 3 (Grx2)
GRX33	Glutaredoxin 3 (Grx3)
GRX	Glutaredoxin
MTGR?	Uncharacterized monothiol glutaredoxin ycf64-like
GRX?	Glutaredoxin-related protein
NrdH	Glutaredoxin-like protein NrdH, required for reduction of Ribonucleotide reductase class Ib
NR	Ribonucleotide reductase of class Ib (aerobic), alpha subunit (EC 1.17.4.1)
Hyp1	Glutathione peroxidase family protein
MTGR	Uncharacterized monothiol glutaredoxin ycf64-like
GR3	Glutaredoxin 3
GR31	Glutaredoxin 3 (Grx1)
GR32	Glutaredoxin 3 (Grx2)
GR33	Glutaredoxin 3 (Grx3)
GR1	Glutaredoxin 1
AUX2	Protein export cytoplasm chaperone protein (SecB, maintains protein to be exported in unfolded state)
AUX3	Glycerol-3-phosphate dehydrogenase [NAD(P)+] (EC 1.1.1.94)
AUX4	tRNA (cytidine(34)-2'-O)-methyltransferase (EC 2.1.1.207) ## TrmL
AUX5	Competence protein F homolog, phosphoribosyltransferase domain
AUX6	Glutathione synthetase (EC 6.3.2.3)

AUX7	Phosphocarrier protein, nitrogen regulation associated
AUX8	ATP/GTP-binding site motif A (P-loop) :BolA-like protein
AUX9	Cell wall endopeptidase, family M23/M37
EnvC/YibP	Periplasmic septal ring factor with murein hydrolase activity EnvC/YibP
RLDP	Rhodanese-like domain protein
RLDP1	FIG136845: Rhodanese-related sulfurtransferase
AUX10	Multidrug resistance protein Atm1
AUX11a	Chaperone protein HscB
AUX11b	Chaperone protein HscA
grlA	Probable monothiol glutaredoxin GrlA
GRR	Glutaredoxin-related protein
AUX12	Peptide chain release factor 1
AUX1	Cell division protein BolA
AUX13	TrkA, Potassium channel-family protein
AUX14	23S rRNA (Uracil-5-) -methyltransferase rumB (EC 2.1.1.-)
AUX15	Oxygen-insensitive NADPH nitroreductase (EC 1.-.-.-)
GR_N?	Glutaredoxin
AUX16	Riboflavin kinase (EC 2.7.1.26)
AUX17	Flavohemoprotein (Hemoglobin-like protein) (Flavohemoglobin) (Nitric oxide dioxygenase) (EC 1.14.12.17)
AUX18	Thiol-disulfide isomerase
AUX19	NAD-dependent glyceraldehyde-3-phosphate dehydrogenase (EC 1.2.1.12)
GR2	Glutaredoxin 2
GRNrdH	Glutaredoxin-like protein NrdH, required for reduction of Ribonucleotide reductase class Ib
YghU	Uncharacterized GST-like protein yghU associated with glutathionylspermidine synthetase/amidase
GTSS	Glutathionylspermidine synthase (EC 6.3.1.8)
GTSA	Glutathionylspermidine amidohydrolase (EC 3.5.1.78)
TrypS	Trypanothione synthetase (EC 6.3.1.9)
Hyp1	Similarity with glutathionylspermidine synthase (EC 6.3.1.8), group 1

Hyp2	Similarity with glutathionylspermidine synthase (EC 6.3.1.8), group 2
